# Orchestration of Endothelial and Osteogenic Marker Expression During Osteogenesis

**DOI:** 10.3390/ijms27114977

**Published:** 2026-05-30

**Authors:** Sydney Chen, Yan Zhao, Nikki Chen, Xiuju Wu, Li Zhang, Zheng Jing, Lei Qi, Xinjiang Cai, Kristina I. Boström, Yucheng Yao

**Affiliations:** 1 Division of Cardiology, David Geffen School of Medicine, UCLA, P.O. Box 951679, Los Angeles, CA 90095-1679, USA; 2 The Molecular Biology Institute, UCLA, Los Angeles, CA 90095-1570, USA

**Keywords:** endothelial cells, differentiation, bone

## Abstract

Vascular endothelial cells (ECs) coordinate with osteogenic processes to establish the specialized vasculature of bone tissue, where endothelial cells and bone cells interact, and bone cells regulate EC proliferation and differentiation. However, it remains unclear how ECs and bone cells are coordinated during early bone formation and whether these interactions differ between endochondral ossification (e.g., femur) and intramembranous ossification (e.g., skull). To address this question, we analyzed endothelial and osteogenic marker expression in the femur and skull between postnatal days 3 and 39. We identified distinct expression patterns of endothelial markers (Endomucin, VE-cadherin and CD31) and osteogenic markers (Osterix, Cbfa1 and BGLP) during osteogenesis in these tissues. In the femurs, endothelial marker expression alternated with the expression of osteogenic markers, suggesting potential reciprocal regulation. In contrast, in the skull, endothelial and osteogenic markers exhibited similar temporal expression patterns without alternation. We also analyzed the expression of VEGF and its receptor FLK1. In the femur, VEGF expression paralleled osteogenic marker expression, whereas in the skull VEGF expression differed from both osteogenic and endothelial marker patterns. Together, these results demonstrate that the coordination of endothelial and osteogenic marker expression, as well as VEGF signaling, differs between endochondral and intramembranous ossification, suggesting distinct modes of interaction between endothelial and bone cells during the formation of long and flat bones.

## 1. Introduction

Endothelial cells (ECs) interact with organ-specific cell types to support normal growth and development [1,2,3]. The formation of functional blood vessels is essential for nutrient delivery, oxygen exchange, and the transport of cells and signaling molecules that regulate systemic homeostasis. ECs play a central role in these processes by forming the inner lining of blood vessels and dynamically interacting with surrounding cells to regulate angiogenesis and vasculogenesis [4]. Disruption of EC differentiation during development can result in structural malformations or developmental arrest, while impaired EC function compromises tissue repair and regeneration [5,6,7].

Bone is a specialized organ in which the vasculature is organized to support growth, remodeling, and mineral metabolism. In long bones such as the femur, central, metaphyseal, and epiphyseal arteries extend into the bone and branch into capillary networks that ultimately drain into the venous system [8]. In contrast, flat bones such as the skull exhibit a distinct vascular architecture, in which surface arteries connect to microvascular networks within the bone marrow and perivascular regions, forming a specialized circulation [9]. These vascular networks are closely associated with osteogenic cells, suggesting coordinated interactions between endothelial and bone cells during bone formation and vascular development.

Bone formation occurs through two major processes: endochondral ossification, which forms most long bones, and intramembranous ossification, which forms flat bones such as those of the skull. Although both processes ultimately generate mineralized bone, they involve distinct cellular and structural pathways during development. Increasing evidence suggests that vascular signals influence osteogenesis and that osteogenic cells can in turn regulate endothelial cell proliferation and differentiation [10]. However, it remains unclear how endothelial and osteogenic processes are temporally coordinated during early bone formation and whether these interactions differ between endochondral and intramembranous ossification.

Markers of endothelial identity, including Endomucin, VE-cadherin, and CD31, have been widely used to characterize vascular development [5], whereas osteogenic markers such as Osterix, Cbfa1 (Runx2), and BGLP (osteocalcin) reflect different stages of osteoblast differentiation and bone formation [11]. In addition, VEGF signaling plays a central role in regulating angiogenesis and vascular development and has also been implicated in coupling vascular growth with bone formation.

In this study, we investigated the coordination between endothelial development and osteogenesis during early postnatal development of long and flat bones. We analyzed the expression of endothelial markers, osteogenic markers, and VEGF signaling components in the femur and skull from postnatal day 3 to day 39. By comparing these two types of ossification, we sought to determine how endothelial and osteogenic processes are temporally organized and whether distinct patterns of coordination exist between endochondral and intramembranous bone formation.

## 2. Results

### 2.1. Expression of Endothelial and Osteogenic Markers During Early Development of Long Bone

**Figure 1:**
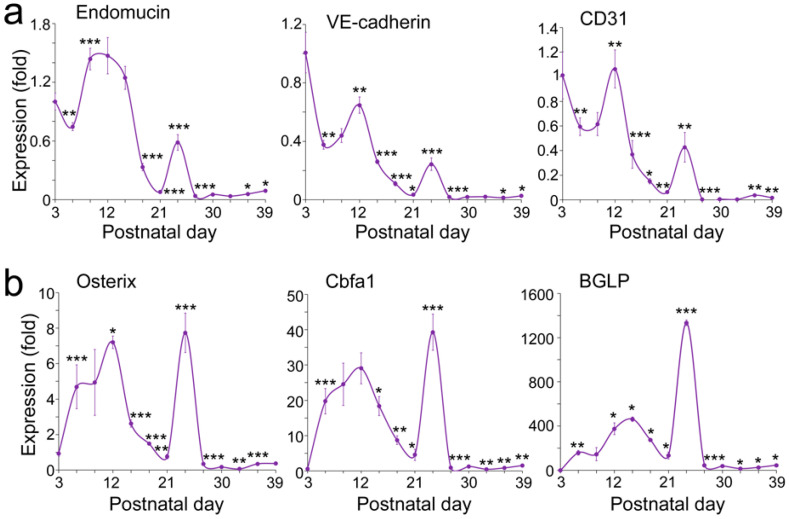
Expression of endothelial and osteogenic markers in femurs during early bone development. Expression of endothelial markers (**a**) and osteogenic markers (**b**) in femurs from postnatal day (P)3 to P39, analyzed by quantitative real-time PCR (n = 8). Gene expression at P3 was used as the reference for fold changes. Each time point from P6 to P39 was compared with the preceding time point, with significant differences indicated (*, *p* < 0.05; **, *p* < 0.01; ***, *p* < 0.001).

To investigate how ECs coordinate with long bone formation, femurs were isolated from mice between postnatal day (P)3 to 39. After removal of the bone marrow, we examined the expression of the endothelial markers Endomucin, VE-cadherin, and CD31. Real-time PCR analysis revealed a distinct temporal pattern of endothelial marker expression characterized by two peaks during this period. The first peak occurred at P12, while a second peak was observed at P24 (Figure 1a). All three endothelial markers exhibited an initial expression peak at P12 and a second peak at P24. Although some early changes were modest in magnitude, the reproducible pattern across markers supports coordinated temporal regulation. At P12, Endomucin expression exceeded its level at P3, whereas VE-cadherin remained lower than their P3 levels (Figure 1a). We next examined the osteogenic markers Osterix, Cbfa1, and BGLP. Osterix and Cbfa1 displayed similar temporal patterns, with low initial expression, a sharp increase from P3 to P12, a decline thereafter, and a second strong peak at P24 (Figure 1b). The expression pattern of BGLP differed slightly, showing a dramatically higher second peak at P24 (Figure 1b). These results suggest that the initial induction of transcription factors Osterix and Cbfa1 during the first peak may mark the onset of osteogenic differentiation, whereas the strong induction of the bone matrix protein BGLP during the second peak likely reflects accelerated bone matrix deposition and maturation.

**Figure 2:**
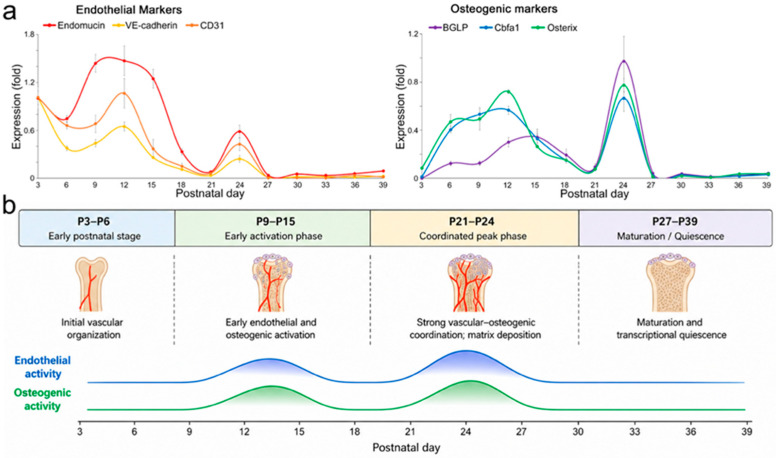
Expression patterns of endothelial and osteogenic markers in femurs during early bone development. (**a**) Comparison of the expression patterns of endothelial markers (Endomucin, VE-cadherin, and CD31, top) and osteogenic markers (Osterix, Cbfa1, and BGLP, bottom). Gene expression at P3 was used as the reference. Each gene is independently normalized to its expression at P3 and plotted to illustrate temporal patterns; values are not intended for direct quantitative comparison between genes. (**b**) Schematic illustration of developmental phases associated with coordinated endothelial and osteogenic gene expression during postnatal femur development.

To better visualize the relationship between endothelial and osteogenic marker expression, we combined the expression curves from Figure 1a,b using arbitrary units (Figure 2). From P3 to P6, osteogenic markers increased markedly with a mild delay in the induction of endothelial markers, suggesting the initiation of tissue transformation and osteogenic differentiation. Between P6 and P18, both endothelial and osteogenic markers showed coordinated waves of induction (Figure 2). Notably, the early induction of osteogenic markers suggested that osteogenic processes may lead endothelial responses during this stage of coordination. The duration of the second peak was similar for both endothelial and osteogenic markers, suggesting a coordinated phase of vascular and skeletal development during long bone growth (Figure 2).

### 2.2. Expression of Endothelial and Osteogenic Markers During Early Development of Flat Bone

**Figure 3:**
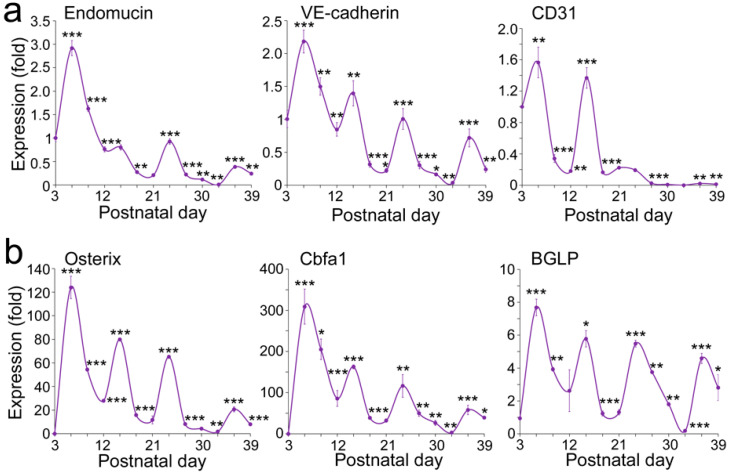
Expression of endothelial and osteogenic markers in the skull during early bone development. Expression of endothelial markers (**a**) and osteogenic markers (**b**) in skull bones from postnatal day (P)3 to P39, analyzed by quantitative real-time PCR (n = 8). Gene expression at P3 was used as the reference for fold changes. Each time point from P6 to P39 was compared with the preceding time point, with significant differences indicated (*, *p* < 0.05; **, *p* < 0.01; ***, *p* < 0.001).

To examine how the ECs coordinated with flat bone formation, skull bones were isolated from mice between postnatal day P3 and P39. The expression of the endothelial markers (Endomucin, VE-cadherin, and CD31) and osteogenic markers (Osterix, Cbfa1, and BGLP) was analyzed by real-time PCR. In contrast to long bones, the endothelial and osteogenic markers in the skull were expressed in similar temporal patterns (Figure 3a,b). From P3 to P6, both endothelial and osteogenic markers increased sharply and reached their highest levels. Following this initial peak, the expression of most markers declined but exhibited additional peaks around P15, P24, and P36 (Figure 3a,b).

**Figure 4:**
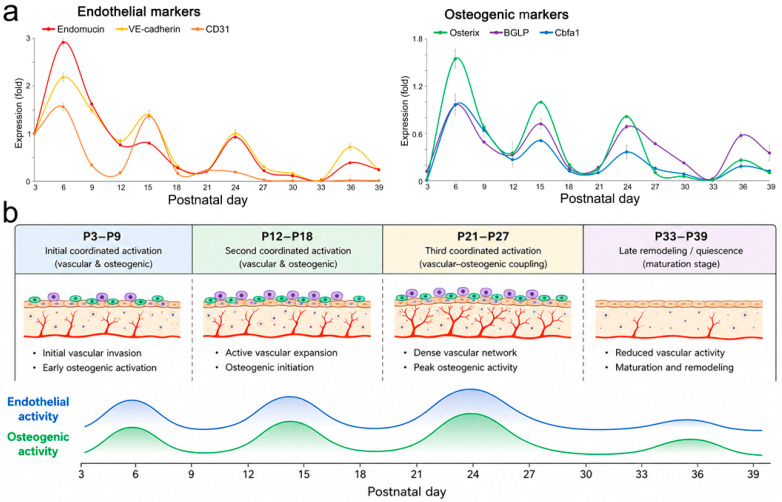
Expression patterns of endothelial and osteogenic markers in the skull during early bone development. (**a**) Comparison of the expression patterns of endothelial markers (Endomucin, VE-cadherin, and CD31, top) and osteogenic markers (Osterix, Cbfa1, and BGLP, bottom). Gene expression at P3 was used as the reference. Each gene is independently normalized to its expression at P3 and plotted to illustrate temporal patterns; values are not intended for direct quantitative comparison between genes. (**b**) Schematic illustration of parallel endothelial and osteogenic regulation during postnatal flat bone development.

To better visualize the relationship between endothelial and osteogenic marker expression, we combined the expression curves from Figure 3a,b using arbitrary units (Figure 4). The combined analysis confirmed that endothelial and osteogenic markers exhibited closely aligned temporal expression patterns during early stages of skull development (Figure 4). Unlike the pattern observed in long bones, there was no early temporal shift between endothelial and osteogenic marker expression in the skull (Figure 4). Instead, both groups of markers appeared to be induced simultaneously during early bone formation (Figure 4).

These results suggest that the coordination between vascular development and osteogenesis during flat bone formation differs from that observed during long bone development, indicating that endothelial–osteogenic interactions may be regulated through distinct mechanisms in intramembranous ossification compared with endochondral ossification.

### 2.3. VEGFA–FLK1 Signaling in Endothelial–Osteogenic Crosstalk During Long and Flat Bone Formation

Our results of coordinated temporal patterns suggest potential endothelial–osteogenic interactions during bone development. To further explore signaling mechanisms that may mediate this relationship, we next examined VEGFA and its receptor FLK1. VEGFA plays a critical role in EC survival, proliferation, and differentiation, and is a key regulator of angiogenesis. In many tissues, VEGFA is primarily produced by non-endothelial cells and acts on VEGF receptors expressed by ECs to coordinate vascular development with tissue growth. To investigate the potential crosstalk between ECs and bone cells during bone formation, we examined the expression of VEGFA and its receptor FLK1 (VEGFR2) in femurs and skull bones from postnatal day P3 to P39.

**Figure 5:**
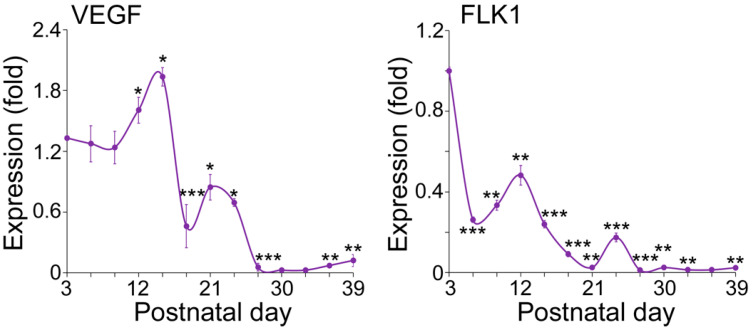
Expression of VEGFA and FLK1 in femurs during early bone development. Expression of VEGFA and FLK1 in femurs from postnatal day P3 to P39, analyzed by quantitative real-time PCR (n = 8). Gene expression at P3 was used as the reference for fold changes. Each time point from P6 to P39 was compared with the preceding time point, with significant differences indicated (*, *p* < 0.05; **, *p* < 0.01; ***, *p* < 0.001).

In the femurs, the expression pattern of VEGFA differed from those of both endothelial and osteogenic markers. VEGFA initial expression was high, similar to that of Endomucin, followed by a decline around P18 and a smaller secondary peak near P21 (Figure 5). In contrast, the expression pattern of FLK1 closely resembled that of endothelial markers such as VE-cadherin and CD31 (Figure 5). These results suggest that VEGFA–FLK1 signaling may contribute to communication between bone cells and ECs during long bone development.

**Figure 6:**
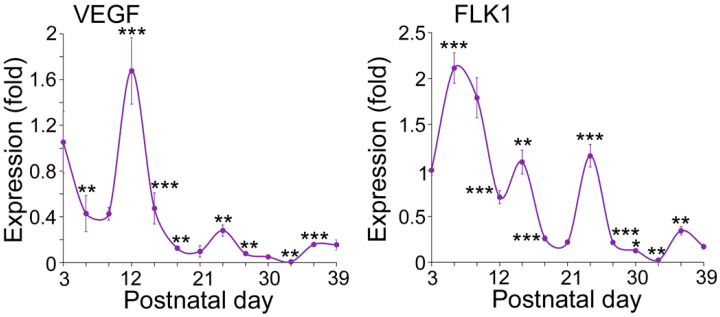
Expression of VEGFA and FLK1 in skull bones during early bone development. Expression of VEGFA and FLK1 in skull bones from P3 to P39, analyzed by quantitative real-time PCR (n = 8). Gene expression at P3 was used as the reference for fold changes. Each time point from P6 to P39 was compared with the preceding time point, with significant differences indicated (*, *p* < 0.05; **, *p* < 0.01; ***, *p* < 0.001).

In skull bones, VEGFA expression exhibited a distinct temporal pattern compared with that observed in the femur. VEGFA levels decreased from the initial high level from P3 to P9 (Figure 6). A pronounced VEGFA peak was observed at P12, which occurred earlier than the second peaks of endothelial and osteogenic markers (Figure 4). This early VEGFA surge may promote vascular development and subsequent bone formation in the skull. As in the femur, the expression of FLK1 in the skull tended to occur prior to VEGF (Figure 6), indicating that ECs remain responsive to VEGF signaling throughout this developmental period.

Together, these results suggest that endothelial differentiation is closely coordinated with osteogenesis during bone development. However, the temporal patterns of VEGFA signaling and endothelial–osteogenic interactions differ between long bone (endochondral ossification) and flat bone (intramembranous ossification). These findings indicate that VEGFA-mediated signaling may contribute to the coordination between ECs and bone cells through mechanisms that vary between different modes of bone formation.

## 3. Discussion

Long bones are formed through endochondral ossification, a process in which cartilage templates are gradually replaced by mineralized bone [12,13,14]. In contrast, flat bones, such as those of the skull, develop through intramembranous ossification, in which mesenchymal cells directly differentiate into osteoblasts without a cartilage intermediate [12,13,14]. Previous studies have shown that vascular invasion plays a critical role in endochondral ossification by promoting the replacement of cartilage with bone tissue. During this process, three major stages of vascularization occur: initial vascular invasion of the diaphysis, capillary invasion of the metaphysis, and vascularization of the epiphysis prior to secondary ossification [8,15]. In contrast, intramembranous ossification is thought to be initiated more independently of early vascular invasion [16,17]. In the present study, we identified distinct temporal expression patterns of endothelial and osteogenic markers in the femur and skull during early postnatal development. These findings suggest that the coordination between endothelial cells (ECs) and bone cells differs between long bone and flat bone formation, likely reflecting the different developmental requirements of endochondral and intramembranous ossification.

Recent studies have shown that ECs in bone can be classified into distinct subtypes based on the expression of Endomucin and CD31. In particular, type H endothelial cells, characterized by high expression of CD31 and Endomucin, are closely associated with osteogenesis and play an important role in supporting bone formation [10,18]. In contrast, type L endothelial cells, which express lower levels of CD31 and Endomucin, are typically found in more mature vascular regions within bone tissue [10,18]. Consistent with these observations, we found that the expression of CD31 and Endomucin peaked on postnatal day 12 in the femur and postnatal day 6 in the skull, coinciding with peaks in osteogenic marker expression. The close temporal association between endothelial and osteogenic marker expression supports the concept that vascular development and bone formation are tightly coupled processes, as blood vessels provide essential nutrients, oxygen, and signaling factors required for osteogenesis.

Interestingly, the temporal patterns of endothelial marker expression differed between the femur and skull. In the femur, the expression of CD31 and Endomucin decreased after postnatal day 27, suggesting a transition toward type L endothelial cells as bone development progresses. In contrast, multiple waves of Endomucin expression were observed in the skull, including a peak around postnatal day 36, when osteogenic markers were also elevated. These findings suggest that vascular remodeling and endothelial–osteogenic interactions may occur in multiple waves during flat bone development. Notably, both long and flat bones exhibited a prominent expression peak around P24. In the femur, this second peak followed an earlier phase in which osteogenic markers increased prior to endothelial markers, whereas at P24 both marker sets increased concurrently. This shift may reflect a transition from early osteogenic differentiation to a phase of coordinated vascular expansion and matrix deposition, in which endothelial and osteogenic processes become more tightly coupled. In the skull, recurrent peaks at P6, P15, P24, and P36 suggest stage-dependent coordination between these processes, with P24 representing one of the more prominent synchronized phases. Although the precise biological events underlying the P24 peak remain to be defined, these findings raise the possibility that this time point corresponds to a key stage of vascular–osteogenic integration during bone maturation.

VEGFA is a key regulator of vascular development and angiogenesis. VEGFA activates its receptor FLK1, which is primarily expressed in endothelial cells, to promote endothelial differentiation and vascular growth [19]. Previous studies have shown that VEGFA is produced by non-endothelial cells in bone tissue, including chondrocytes and osteogenic progenitors, where it acts as a signaling molecule to coordinate vascular invasion with bone formation [20,21]. In this study, we observed that FLK1 expression peaked prior to induction of VEGFA, suggesting that VEGFA signaling may initiate or facilitate subsequent vascular and osteogenic processes. Moreover, the temporal expression patterns of VEGFA differed from those of both endothelial and osteogenic markers in the femur and skull. These observations suggest that VEGFA-mediated signaling may occur during transitional phases between waves of vascular and bone growth.

The temporal expression patterns observed in this study suggest that endothelial and osteogenic programs during bone development may be coordinated through dynamic, potentially oscillatory regulatory mechanisms. In long bones, both endothelial and osteogenic markers exhibited distinct waves of expression characterized by two major peaks during postnatal development, whereas in flat bones, these markers displayed synchronized, multi-peak patterns. Notably, the early induction of osteogenic transcription factors preceded or coincided with endothelial marker expression, while a later coordinated peak corresponded to phases of active matrix deposition and vascular expansion. While the dependence of endochondral ossification on vascular invasion and the highly vascularized nature of intramembranous ossification are well established, our findings extend this paradigm by revealing stage-specific temporal coordination between endothelial and osteogenic gene expression programs.

Oscillatory regulatory systems are increasingly recognized as mechanisms for coordinating complex biological processes [22]. For example, feedback loops involving BMP ligands and their extracellular inhibitors have been shown to generate oscillatory expression patterns that organize endothelial behavior during vascular growth, while the p53–Mdm2 negative feedback system demonstrates how oscillations can temporally orchestrate downstream cellular responses [23,24]. By analogy, the repeated peaks in endothelial and osteogenic gene expression observed here raise the possibility that bone formation may also be governed by feedback-driven regulatory networks that coordinate angiogenesis with osteogenic differentiation. Such regulation could synchronize vascular expansion with sequential phases of osteoblast differentiation and matrix production during skeletal growth. Differences in temporal alignment between endothelial and osteogenic markers in long versus flat bones further suggest that these dynamics may be modulated according to the distinct developmental programs underlying endochondral and intramembranous ossification.

Although *Runx2* is widely regarded as an upstream regulator of osteogenesis [25,26], its mRNA expression did not precede other osteogenic markers in our dataset. This may reflect the temporal resolution of our study, which begins at P3 and therefore does not capture earlier developmental stages when initial *Runx2* induction may occur. In addition, our analysis measures transcript levels in whole bone, where cell heterogeneity may obscure transient or cell-specific changes. Furthermore, RUNX2 activity is extensively regulated at the post-transcriptional and post-translational levels, and therefore may not be directly inferred from mRNA expression alone. In addition, the osteogenic markers selected in this study, Cbfa1, Osterix, and BGLP, represent key stages of osteogenic progression, including early transcriptional commitment Cbfa1, osteoblast differentiation Osterix, and matrix maturation BGLP. Our goal was to capture the temporal dynamics of representative markers across distinct phases of osteogenesis rather than to provide a comprehensive profiling of all osteogenic genes. Additional markers such as Alpl, Bmp2, and others could further complement the analysis.

This study provides a systematic analysis of temporal gene expression patterns during bone development, while several considerations should be noted. The analysis is based on mRNA expression, which captures transcriptional dynamics underlying endothelial and osteogenic coordination, although it does not directly assess protein abundance or activity. In addition, gene expression was measured in whole bone tissue, providing an integrated view of tissue-level dynamics, but potentially averaging cell-type–specific or transient changes. Furthermore, murine bone lacks fully developed Haversian systems present in human cortical bone, which may influence aspects of vascular organization and remodeling. Despite these considerations, the consistent temporal patterns observed across endothelial and osteogenic markers offer insight into coordinated developmental processes. These findings establish a framework for future studies to investigate protein-level regulation, functional mechanisms, and validation in human systems.

Together, our findings indicate that endothelial differentiation and osteogenesis are closely coordinated during early bone development. However, the temporal patterns of endothelial–osteogenic interaction differ between long bones and flat bones, reflecting the distinct mechanisms underlying endochondral and intramembranous ossification. Manipulating VEGFA signaling or endothelial cell populations in vivo may help determine how changes in endothelial subtype balance influence bone growth and patterning. In addition, advanced imaging approaches such as three-dimensional micro-computed tomography with vascular contrast agents could provide valuable insights into the spatial dynamics of vascular invasion and remodeling during bone development.

## 4. Methods

### 4.1. Animals

Wild-type mice on a C57BL/6J background were originally obtained from The Jackson Laboratory and subsequently bred in-house at the Division of Laboratory Animal Medicine (UCLA). Mice were maintained on a standard chow diet (Diet 8604, Harlan Teklad Laboratory, Madison, WI, USA) with free access to food and water. All procedures were conducted in accordance with the Guide for the Care and Use of Laboratory Animals (NIH Publication No. 85-23, revised 1996) and were approved by the institutional animal care and use committee.

A total of 124 mice were used. Sample size was determined based on a priori power calculations informed by our previous studies [27]. Both male and female mice were included in each group. Tissue collection was performed at the time points described in the main text. Mice were euthanized using methods consistent with institutional and national guidelines. All procedures were approved by the Institutional Animal Care and Use Committee (IACUC) of the University of California, Los Angeles.

### 4.2. RNA Isolation and Quantitative Real-Time PCR

For RNA isolation, bones were fully dissected and cleared of surrounding soft tissue prior to processing. Bone samples were lysed and homogenized by mechanical homogenization. Total RNA was isolated using the RNeasy Mini Kit (Qiagen, Hilden, Germany) according to the manufacturer’s instructions [27]. RNA concentration and purity were assessed using a microvolume spectrophotometer (Epoch, BioTek, Winooski, VT, USA).

Complementary DNA (cDNA) was synthesized from 1 µg of total RNA using the High-Capacity cDNA Reverse Transcription Kit (ThermoFisher Scientific, Waltham, MA, USA) following the manufacturer’s protocol. Quantitative real-time PCR was performed using TaqMan™ Universal Master Mix II (ThermoFisher Scientific, Waltham, MA, USA) on a ViiA7 Real-Time PCR System (Applied Biosystems, Waltham, MA, USA) as previously described [12]. Gene expression assays were obtained from TaqMan^®^ Gene Expression Assays (Applied Biosystems); assay IDs are as follows: *Endomucin* (Mm00497499_m1), *CD31* (Mm01242582_m1), *VE-cadherin* (Mm00486938_m1), *Osterix* (Mm00504574_m1), *Cbfa1* (Mm00501584_m1), *BGLP* (Mm00515388_m1), *FLK1* (Mm01222419_m1), and *VEGF* (Mm00437306_m1). Gene expression levels were normalized to Gapdh, selected based on our previous study [27]. Relative expression was calculated using the ΔCt method, as no external control condition was included. Each data point represents an independent biological replicate (n = 8 animals per time point), with real-time PCR performed in technical triplicates.

### 4.3. Statistical Analysis

Data were analyzed using a one-way analysis of variance (ANOVA) with Benjamini–Hochberg (BH) correction. Statistical analyses were performed using GraphPad InStat^®^ version 3.0 (GraphPad Software). Data are presented as mean ± SD. A *p* value < 0.05 was considered statistically significant. All experiments were repeated at least three times.

## Data Availability

The data presented in this study are available on request from the corresponding author due to institutional restrictions and ongoing research.
